# Antimicrobial activity of some plant materials used in Armenian traditional medicine

**DOI:** 10.1186/s12906-017-1573-y

**Published:** 2017-01-17

**Authors:** Mikayel Ginovyan, Margarit Petrosyan, Armen Trchounian

**Affiliations:** Department of Microbiology, Plants and Microbes Biotechnology, Faculty of Biology, Yerevan State University, 1 Alex Manoogian Str., Yerevan, 0025 Armenia

**Keywords:** Plant material, Armenian folk medicine, Antibiotic resistance, Crude extract, Antimicrobial activity, Minimum bactericidal concentration, Acetone

## Abstract

**Background:**

Antibiotic resistance has become one of the major problems facing humanity. The need for new antimicrobials has been increased dramatically. Plants are considered as one of the most promising sources for new antimicrobials discovery. Despite relatively small area, Armenia has large diversity of flora with many endemic species. In Armenian folk medicine plant materials have been used to treat various microbial diseases since ancient times. The goal of our research was to evaluate antimicrobial efficiency of different parts of five wild plants species which are commonly used in Armenian traditional medicine.

**Methods:**

Plant crude extracts were obtained with maceration technique using five solvents separately: distilled water, methanol, chloroform, acetone, and hexane. Agar well diffusion assay was used for initial evaluation of antimicrobial properties of plant materials against five bacterial and two yeast strains. Minimum inhibitory concentrations of the most active plant parts were determined by broth microdilution method.

**Results:**

Crude extracts of all five tested plants expressed antimicrobial activity against at least four test strains at 500 μg ml^−1^ concentration. Minimum inhibitory and bactericidal/fungicidal concentrations of selected plant parts were determined. Crude acetone and hexane extracts of *Hypericum alpestre* and acetone extract of *Sanguisorba officinalis* inhibited the growth of *P. aeruginosa* even at 64 μg ml^−1^ concentration. Chloroform and acetone extracts of *Sanguisorba officinalis* exhibited cidal activity against *P. aeruginosa* till 256 μg ml^−1^. Acetone was the most effective solvent for solubilizing antimicrobial compounds for almost all tested plant materials.

**Conclusions:**

Thus, antimicrobial activity of some medicinal plants used in Armenian traditional medicine was evaluated. Some of the plants had rather low minimum bacteriostatic/bactericidal concentrations and therefore they have prospective for further more inclusive studies.

**Electronic supplementary material:**

The online version of this article (doi:10.1186/s12906-017-1573-y) contains supplementary material, which is available to authorized users.

## Background

The prevalence of microbial infectious diseases and their complications are continuously increasing throughout the world mainly due to microbial drug resistance toward commonly used antimicrobials [1]. Antibiotic resistance has become one of the major problems of humanity since late 20^th^ century. The need for new antimicrobials, which could effectively fight against resistant microbes, has tremendously increased. Traditional approaches to find new antimicrobial drugs are not sufficiently successful anymore due to the rapid resistance development against them [2]. Consequently, it is very important to find new approaches of antimicrobial compounds discovery. Plant materials are demonstrated to be one of the most promising sources [2–4]. Plant-derived antimicrobials are also considered to be safer compared with synthetic compounds because of their natural origin [5, 6]. It is well known that about quarter part of current medications is derived from compounds of plant origin [1, 7]. Plant-derived compounds could have other target sites than traditional antimicrobials and subsequently having different mechanisms of action against microbes [6, 8–10].

Plant secondary metabolites are mostly responsible for their antimicrobial activity [4]. Major groups of phytochemicals which possess antimicrobial properties are phenolics and polyphenols (flavonoids, quinones, tannins, coumarins), terpenoids, alkaloids, lectins and polypeptides [3, 4, 6]. There are several mechanisms that underlie antimicrobial action of plant-derived compounds. Phytochemicals can act by disrupting microbial membranes (carvacrol, thymol, eugenol, *etc.*) or impairing cellular metabolism (cinnamaldehyde). They can also control biofilm formation (trans-cinnamaldehyde, carvacrol, thymol, geraniol, *etc.*). Plant antimicrobials can inhibit bacterial capsule production (salicylic acid and its derivatives). Some plant compounds can attenuate bacterial virulence by controlling quorum-sensing. Another mechanism of plant metabolites’ antimicrobial action is the reduction of microbial toxin production (dihydroisosteviol, RG-tannin, *etc.*) [4, 6]. Plant metabolites can also act as resistance-modifying agents (RMAs). Nowadays RMAs are considered as one of the most prospective ways to combat bacterial resistance. Some studies already showed that plant-derived compounds can enhance therapeutic effect of antibiotics acting as RMAs (nerolidol, bisabolol, apritone, *etc.*) [2, 6].

Plant species are estimated to be around 250–500 thousands [3]. However, only a small part of them were investigated for antimicrobial activity [4, 10, 11]. People started to use plant materials to treat infectious diseases since ancient times even without any knowledge on their causative agents [12]. Nowadays, herbs continue to be used in traditional medicine to heal various infectious conditions in many countries, including Armenia. Moreover, in the last decades this tendency has increased [13].

Despite relatively small area, Armenia has large diversity of flora with many endemic species. This diversity is mainly due to well-defined vertical zonation, which provides the variety of weather conditions in the country. Since ancient times Armenia has been well-known for its herbal medicine. At the present time the production of herbal teas, essential oils, and powders is well established [14]. There were some studies which tried to evaluate antimicrobial properties of plants from Armenian region. Nevertheless, they were mainly focused on particular plant species or genera. There is lack of massive studies to evaluate medicinal plants of the region for their antimicrobial properties.

Three main approaches can be used to select plants for antimicrobial testing. First approach is to find antimicrobial activity in plants used in folk medicine. The second one is to search antimicrobial properties within plants typical for a particular region or country. And the third approach is to explore antimicrobial efficiency of various plants against a particular microbe [12]. We merged the first two approaches in order to enhance the possibility of finding plants with high antimicrobial efficiency in Armenia.

Our goal was to investigate antimicrobial activity of crude aqueous, methanol, chloroform, acetone, and hexane extracts of five Armenian herbs.

## Methods

### Collection, identification and drying of plant materials

Plants species have been chosen based on their use in folk medicine. Particularly, we selected plants which have been used for the treatment of purulent wounds, burns, infections and inflammations. In addition, attention was given to plants typical for Armenian region. The collection of plant materials was done in dry and sunny days. Leaves, flowers and whole plants were harvested in the flowering period. Whereas, fruits, seeds and roots were harvested after maturation according to recommendations [15].

Identification of plant materials was done at the Department of Botany and Mycology, Yerevan State University (Armenia), by Dr. N. Zakaryan and Dr. A. Poghosyan. Immediately after harvesting of plant materials, they were thoroughly washed with distilled water. Then they were placed in drying room under shadow provided with dry air conditioning for ten days. Afterwards, the dried plant materials were fine grounded with a homogenizer (Homogenizer type MPW-302, Poland) and stored in hermetically sealed glass jars at room temperature.

### Collected plant materials

Collected plant materials and details are listed in Additional file 1: Table S1. Voucher specimens of all plants were deposited to the Herbarium of Yerevan State University (Yerevan, Armenia) where serial numbers were given; *Agrimonia eupatoria* L. (ERCB 13207), *Hypericum alpestre* subsp. *polygonifolium* (Rupr.) Avet. & Takht. (ERCB 13206), *Lilium armenum* (Miscz. ex Grossh.) Manden. (ERCB 13209), *Rumex obtusifolius* L. (ERCB 13208), *Sanguisorba officinalis* L. (ERCB 13205) (See Table 1).

**Table 1:** The list of the tested plant species names with common names, family names, tested parts and traditional uses in Armenian folk medicine

Plant name^a^	Common name	Family	Part tested	Traditional uses^b^
*Agrimonia eupatoria* L. (ERCB 13207) ^c^	Common agrimony	Rosaceae	Whole plant	Hepatitis, nephritis, stomatitis, yellow fever, purulent wounds
*Hypericum alpestre* subsp. *polygonifolium* (Rupr.) Avet. & Takht. (ERCB 13206)	Hypericum	Hypericaceae	Aerial part	Pneumonia, wounds, hepatitis, cholecystitis gastrointestinal inflammation, nephritis, skin diseases
*Lilium armenum* (Miscz. ex Grossh.) Manden. (ERCB 13209)	Unknown	Liliumceae	Leaf with stalk, bulb	Whooping cough, purulent wounds, burns, leprosy, fungal diseases, mastitis, cystitis
*Rumex obtusifolius L.* (ERCB 13208)	Broad-leaved dock	Polygonaceae	Leaf, root, inflorescence seed	Infectious diseases, skin rash, mucosal inflammation
*Sanguisorba officinalis* L. (ERCB 13205)	Great burnet	Rosaceae	Aerial part, root	Tonsillitis, purulent wounds, inflammations, skin infections, dysentery, typhoid fever, trichomoniasis, stomatitis, gingivitis, flu

^a^The plants names have been checked with http://www.theplantlist.org/

^b^In this section there are presented only such traditional uses which can imply the presence of antimicrobial compounds [20, 21]

^c^Voucher specimen serial number

### Preparation of plant crude extracts

Plant materials were extracted by maceration technique with five solvents as follows: distilled water, methanol (98%), chloroform (99%), acetone (99.8%), and hexane (97%) (Sigma-Aldrich). Grinded plant materials were soaked with each solvent separately at 10:1 solvent-to-sample ratio (v/w). The mixture was thoroughly vortexed for one minute and left in refrigerator at 5^0^C for 24 h according to the method developed by Rojas et al. [13]. Then, the mixtures were filtered through Whatman filter paper (11 μm pore size). The filtrates were placed in a vacuum chamber (BOV-50 V vacuum drying oven, Biobase Meihua Trading, China) at 40^0^C temperature for drying. Further, fresh solvents were added to the residue at the same ratio and left at 5^0^C for next 24 h. This step was repeated three times in order to achieve maximal extraction of active compounds. Dried crude extracts were weighed and kept at 5^0^C till further use (not more than one month).

### Used microorganisms and growth conditions

Following microorganisms were used as test strains: bacteria *Escherichia coli* VKPM-M17 (Russian National Collection of Industrial Microorganisms, Moscow, Russia), *Pseudomonas aeruginosa* GRP3 (Soil and Water Research Institute, Iran), *Bacillus subtilis* WT-A1, isolated from metal polluted soils of Kajaran, Armenia, *Salmonella typhimurium* MDC 1754 and *Staphylococcus aureus* MDC 5233 (Microbial Depository Center, Armbiotechnology Scientific and Production Center, Yerevan, Armenia), yeasts *Candida albicans* WT-174 isolated from infected vaginal microbiota of hospitalized patients, and *Candida guilliermondii* HP-17 (Department of Botany and Mycology, YSU, Yerevan, Armenia).

Mueller-Hinton broth medium (MHB) and Mueller-Hinton agar medium (MHA) (Liofilchem, Italy) were used for cultivation of bacteria. Mueller-Hinton (Broth) Agar + 2% Glucose medium and 0.5 μg ml^−1^ Methylene Blue Dye (GMB) were used for cultivation of yeasts [16].

### Preparation of stock solutions

The stock solutions of the samples were prepared by dissolving crude dried extracts in pure dimethyl sulfoxide (DMSO) (Sigma-Aldrich) (in order to avoid sterile filtration), with the exception of aqueous extracts, which were dissolved in sterile distilled water and filtered through 0.22 μm sterile filter (Millipore).

### Initial evaluation of plants’ antibacterial and anti-yeast activity

The antimicrobial activity of the samples were initially evaluated by modified agar well diffusion assay [17]. Growth medium (25 ml) was poured into Petri dishes at 50–70 °C and it was left to solidify under ultraviolet (UV) light (265 nm wavelength) for 15 min. Subsequently, a sterile cotton swab was dipped into overnight bacterial or yeast suspensions of indicator strains (adjusted to turbidity of 0.5 McFarland Standard). An agar plate was inoculated by evenly streaking cotton swab over the agar medium. Then wells with a diameter of 8 mm were cut in the medium with a sterile cork borer. Stock solutions of the samples were diluted in sterile distilled water to get 500 μg ml^−1^ concentrations. The tested samples and controls (100 μl) were dispensed into the wells. The plates were incubated at 37 °C for 24 h. Then the diameters of growth inhibition zones around the wells were measured. Following control agents were used: positive control agents - gentamicin (10 μg ml^−1^) (for bacteria) and nystatin 20 μg ml^−1^ (for yeasts); negative control agent - 5% DMSO.

### Determination of MIC values

Determination of Minimum inhibitory concentrations (MIC) of plant crude extracts was done using broth microdilution method [18]. Sample concentration range was prepared from the stock solutions by two-fold dilutions in sterile broth. Six dilutions of the samples ranging from 32 to 1024 μg ml^−1^ were tested. The inoculums of test strains prepared from fresh overnight cultures were adjusted to 0.5 McFarland standard, which equals to 1-2 × 10^8^ CFU ml^−1^ for bacteria and 1-5 × 10^6^ CFU ml^−1^ for yeasts. The inoculums then were diluted in 1:100 ratio in case of bacteria and in 1:10 ratio for yeasts in order to get 1-5× 10^5^ CFU ml^−1^ concentrations. The 75 μl volume of samples were poured to each well of 96-well microplate (Sarstedt, Germany). Then 75 μl volume of test strain suspensions were added to them. The highest dilution of samples without visible growth after 24 h incubation at 37^0^C was considered as MIC.

For this assay the positive control agents were gentamicin (range: 0.03–2 μg ml^−1^) for bacteria and nystatin (range: 0.06–4 μg ml^−1^) for yeasts. Whereas, 1% DMSO (which was present in extracts with 1024 μg ml^−1^ concentration) and broth were used as negative controls. All dilutions of crude extracts were cultured in agar media for sterility test.

### Determination of MBC/MFC values

Minimum bactericidal/fungicidal concentrations (MBC/MFC) of the plant crude extracts were determined by sub-culturing the samples (5 μl) taken from the wells without growth during MIC determination on agar medium. The lowest concentration of crude extracts with the absence of growth after 24 h incubation at 37 ^0^C was considered as MBC/MFC.

### Data processing

All experiments were independently repeated three times. Obtained data were processed; standard deviations were calculated using GraphPad Prism 5.03 (GraphPad Software, Inc.; USA) software.

## Results and dicussion

### Screening of plant materials and selection of the most prospective plants

Five plant species: *Sanguisorba officinalis*, *Rumex obtusifolius*, *Hypericum alpestre*, *Lilium armenum* and *Agrimonia eupatoria* (Additional file 1: Table S1)*,* were chosen based on initial *in vitro* evaluation of antimicrobial activities of different parts of 28 wild plant species against five bacterial and two yeast strains. All plant materials are widely used in Armenian traditional medicine.

In order to select the most active parts of five plant species, agar well diffusion assay was used. We used 500 μg ml^−1^ concentration of crude extracts (50 μg dry material for each well) for agar well diffusion assay according to recommendations [12, 19]. Rios and Recio (2005) in their review stated that using crude extracts of plant materials with concentrations above 1000 μg ml^−1^ in antimicrobial screening protocols should be avoided, because using high concentrations of plant crude extracts can bring to false positive results [19].

According to the obtained data all plant parts possessed antimicrobial activity against at least three tested strains (see Table 2). In order to select prospective plant parts for further comprehensive studies we used two main criteria. At first, we paid attention to plant extracts with the highest growth inhibition zones. We also gave a priority toward the samples which had broad spectrum of action against various microbial groups (Gram-positive, Gram-negative, endospore forming bacteria and yeast). Thus, according to data obtained (see Table 2) following plant parts were chosen for further more profound investigation: *Sanguisorba officinalis* (aerial part), *Rumex obtusifolius* (seed), *Hypericum alpestre* (aerial part), *Lilium armenum* (bulb), and *Agrimonia eupatoria* (whole plant).

**Table 2:** Antibacterial and anti-yeast activity of the tested plant extracts determined by agar well diffusion assay

Plant species	Part tested	Extract	Diameter of growth inhibition zone with standard deviation (mm)						
			*SA* ^*a*^	*BS*	*PA*	*EC*	*ST*	*CG*	*CA*
*Agrimonia eupatoria*	Whole plant	Water	–	–	–	9 ± 0.6	–	–	–
		Methanol	10 ± 0.6	11 ± 0.6	11 ± 0.6	11 ± 0.6	–	11 ± 0.6	–
		Chloroform	9	11 ± 0.6	10 ± 0.6	10 ± 0.6	11 ± 0.6	12 ± 0.6	9 ± 0.6
		Acetone	12 ± 0.6	10 ± 0.6	11 ± 0.6	10 ± 0.6	10 ± 0.6	12 ± 1	9 ± 0.6
		Hexane	13 ± 0.6	10 ± 0.6	10 ± 0.6	–	9 ± 0.6	9 ± 0.6	–
*Hypericum alpestre*	Aerial part	Water	–	–	11 ± 0.6	–	–	–	–
		Methanol	10 ± 0.6	11 ± 0.6	18 ± 0.6	10 ± 0.6	–	–	–
		Chloroform	13 ± 0.6	12 ± 0.6	23 ± 1	–	–	–	–
		Acetone	15 ± 0.6	13 ± 0.6	21 ± 0.6	–	10 ± 0.6	9 ± 0.6	–
		Hexane	17 ± 0.6	16 ± 0.6	21 ± 0.6	–	–	–	–
*Lilium armenum*	Stalk with leaf	Water	–	–	10 ± 0.6	–	–	–	–
		Methanol	9 ± 0.6	–	13 ± 0.6	–	–	–	–
		Chloroform	9 ± 0.6	–	10 ± 0.6	–	–	–	–
		Acetone	10 ± 0.6	9	11 ± 0.6	–	–	–	–
		Hexane	9 ± 0.6	–	12 ± 0.6	–	–	–	–
*Lilium armenum*	Bulb	Water	–	–	9 ± 0.6	–	–	–	–
		Methanol	9 ± 0.6	9 ± 0.6	11 ± 0.6	9 ± 0.6	–	–	–
		Chloroform	9 ± 0.6	–	13 ± 0.6	–	9 ± 0.6	–	–
		Acetone	10 ± 0.6	9 ± 0.6	11 ± 0.6	9 ± 0.6	9 ± 0.6	–	–
		Hexane	–	–	11 ± 0.6	–	9 ± 0.6	–	–
*Rumex obtusifolius*	Leaf	Water	–	–	–	–	–	–	–
		Methanol	9 ± 0.6	–	–	–	10 ± 0.6	14 ± 0.6	–
		Chloroform	11 ± 0.6	–	10 ± 0.6	–	10 ± 0.6	9 ± 0.6	–
		Acetone	11 ± 0.6	–	12 ± 0.6	–	10 ± 0.6	12 ± 0.6	–
		Hexane	9 ± 0.6	–	11 ± 0.6	9 ± 0.6	–	–	–
*Rumex obtusifolius*	Root	Water	–	–	11 ± 0.6	–	–	–	–
		Methanol	10 ± 0.6	–	10 ± 0.6	–	–	–	9 ± 0.6
		Chloroform	11 ± 0.6	–	10 ± 0.6	–	9 ± 0.6	12 ± 0.6	9 ± 0.6
		Acetone	10 ± 0.6	9 ± 0.6	12 ± 0.6	–	–	13 ± 0.6	9 ± 0.6
		Hexane	9 ± 0.6	–	–	–	–	12 ± 0.6	–
*Rumex obtusifolius*	Inflorescence	Water	–	–	–	–	–	–	–
		Methanol	–	9 ± 0.6	9 ± 0.6	10 ± 0.6	–	–	–
		Chloroform	–	9 ± 0.6	9 ± 0.6	9 ± 0.6	–	–	–
		Acetone	11 ± 0.6	11 ± 0.6	9 ± 0.6	10 ± 0.6	9 ± 0.6	9 ± 0.6	–
		Hexane	9 ± 0.6	9 ± 0.6	10 ± 0.6	–	–	–	–
*Rumex obtusifolius*	Seed	Water	9 ± 0.6	–	10 ± 0.6	–	–	–	–
		Methanol	12 ± 0.6	11 ± 0.6	10 ± 0.6	11 ± 0.6	12 ± 0.6	10 ± 0.6	–
		Chloroform	–	9 ± 0.6	9 ± 0.6	–	–	–	–
		Acetone	12 ± 0.6	12 ± 0.6	10 ± 0.6	10 ± 0.6	12 ± 0.6	10 ± 0.6	–
		Hexane	9 ± 0.6	9 ± 0.6	10 ± 0.6	–	–	–	–
*Sanguisorba obtusifolius*	Aerial part	Water	–	–	10 ± 0.6	–	9 ± 0.6	–	–
		Methanol	12 ± 0.6	11 ± 0.6	10 ± 0.6	10 ± 0.6	12 ± 0.6	9 ± 0.6	10 ± 0.6
		Chloroform	10 ± 0.6	9 ± 0.6	10 ± 0.6	–	–	12 ± 0.6	11 ± 0.6
		Acetone	13 ± 0.6	13 ± 0.6	12 ± 1	12 ± 0.6	11 ± 0.6	10 ± 0.6	10 ± 0.6
		Hexane	11 ± 0.6	10 ± 0.6	10 ± 0.6	9 ± 0.6	–	10 ± 0.6	9 ± 0.6
*Sanguisorba officinalis*	Root	Water	–	–	–	–	–	–	–
		Methanol	10 ± 0.6	–	10 ± 0.6	10 ± 0.6	–	10 ± 0.6	10 ± 0.6
		Chloroform	–	–	–	–	–	10 ± 0.6	11 ± 0.6
		Acetone	10 ± 0.6	10 ± 0.6	10 ± 0.6	10 ± 0.6	–	10 ± 0.6	10 ± 0.6
		Hexane	–	–	–	–	–	–	10 ± 0.6
	PC^b^		20 ± 0.6	30 ± 1	28 ± 1	19 ± 0.6	23 ± 0.6	24 ± 0.6	23 ± 0.6

^a^Used test strains: *Escherichia coli* WKPM-M17 (EC), *Pseudomonas aeruginosa* GRP3 (VKPH B-82–5) (PA), *Bacillus subtilis* WT-A1 (BS)*, Salmonella typhimurium* WDCM 1754 (ST)*, Staphylococcus aureus* WDCM 5233 (SA), *Candida albicans* 174 (CA)*, Candida guilliermondii* HP-17 (CG)

^b^PC – positive control (gentamicin (10 μg ml^−1^) (for bacteria), nystatin 20 μg ml^−1^ (for yeasts))

All experiments were independently repeated three times. Average means with standard deviations are represented. Standard deviations were calculated with GraphPad Prism 5.03 (GraphPad Software, Inc.; USA) software

All tested parts of *Rumex obtusifolius* demonstrated substantial antimicrobial activity with some differences (see Table 2). For example, only root extracts exhibited antimicrobial activity against *C. albicans.* In turn they did not inhibit the growth of *E. coli*, whereas its other parts did. *B. subtilis* was more sensitive to crude extracts of inflorescence and seed of *Rumex obtusifolius*. In contrast, tested yeast strains were more sensitive to leaf and root extracts of this plant. Acetone and methanol extract of *Rumex obtusifolius* seeds showed the highest antimicrobial activity against all tested strains except *C. albicans.* Consequently, we have chosen this part for further study. Other parts of *Rumex obtusifolius* had less efficiency compared to the seeds. Although root crude extracts had lower activity than seed extracts, however, they were also interesting due to their activity against *C. albicans.*

Aerial part of *Lilium armenum* inhibited the growth of only three bacterial test strains. Meanwhile, its bulb extracts exhibited activity against all tested bacterial strains. Moreover, growth inhibition zones of the bulb extracts exceed zones, induced by the aerial part. Therefore, the bulb part of *Lilium armenum* was chosen for further studies. This plant is also considered interesting, as it is native to Armenia, where it is widely used in folk medicine. However, to this date there have been no studies about its antimicrobial properties [20, 21].

Among all the tested plant materials the largest zones of inhibition were induced by *Hypericum alpestre* extracts (see Table 2). They exhibited activity against almost all test strains with the exception of *C. albicans* and *S. typhimurium.* Even though other species of the genera *Hypericaceae* were widely investigated and their high antimicrobial activity has already been shown in the literature [22, 23], there was no information about *Hypericum alpestre’s* antimicrobial properties. However, it was the most commonly used species in traditional medicine within the genera in the region from where it was collected.

Even though the use of the roots of *Sanguisorba officinalis* is more common in traditional medicine [20, 21], the obtained data demonstrated higher antimicrobial activity of the aerial part compared to the root. Acetone and methanol extracts of *Sanguisorba officinalis’s* aerial part inhibited the growth of the tested strains whereas crude extracts of the root did not show activity against *S. typhimurium* at tested concentrations. Therefore, we selected the aerial part for further research. Crude extracts of *Agrimonia eupatoria* also inhibited the growth of all tested bacterial and yeast strains (see Table 2). Although *Sanguisorba officinalis* and *Agrimonia eupatoria* were already investigated in many research works and their high antimicrobial activity was shown [24–26], it was interesting to study these plants in order to find out any possible differences between the activity of same plant species from various geographical areas.

We compared the activities of the tested plant materials against Gram-negative and Gram-positive bacteria based on agar well diffusion assay (Additional file 1: Table S2). Crude extracts of *Hypericum alpestre* were more active against Gram-positive bacteria. In case of *Rumex obtusifolius, Lilium armenum, Agrimonia eupatoria* and *Sanguisorba officinalis* in general similar activity was observed against both Gram-negative and Gram-positive bacteria.

Thus, screening of different parts of plant species used in Armenian folk medicine allowed us to evaluate their antimicrobial properties at 500 μg ml^−1^ concentration and choose the most active plant parts for further studies. On the other hand, the obtained results enabled the evaluation of comparative effectiveness of five different solvents for their ability to solubilize antimicrobial compounds from plant materials.

### Determination of MIC values of selected plant materials

MIC values of the selected five plant parts: *Lilium armenum* (bulb), *Rumex obtusifolius* (seed), *Hypericum alpestre* (aerial part), *Agrimonia eupatoria* (whole plant) and *Sanguisorba officinalis* (aerial part) extracted with five solvents were determined (see Table 3). According to collected data MIC values of the tested extracts generally varied within the range from 64 μg ml^−1^ to 1024 μg ml^−1^. The MIC values of aqueous, chloroform and hexane extracts for almost all tested plants were relatively high. Therefore, they cannot be considered for any further practical use based on their weak activity. The exceptions were chloroform and hexane extracts of *Hypericum alpestre* which had low (128 μg ml^−1^) MIC values against *S. aureus*. Moreover, hexane extract of this plant continued to be active till 64 μg ml^−1^ concentration against *P. aeruginosa.* Another exception was hexane extract of *Agrimonia eupatoria* with 128 μg ml^−1^ MIC value against *P. aeruginosa*. In contrast, MICs of acetone and methanol extracts of the tested plant materials had fairly low values against some test strains. For instance, methanol and acetone extracts of *Hypericum alpestre* had 128 μg ml^−1^ and 64 μg ml^−1^ MIC values respectively against *P. aeruginosa.* The MIC of acetone extract of the same plant against *S. aureus* and *B. subtilis* was 128 μg ml^−1^. The MIC value of acetone extract of *Sanguisorba officinalis* against *P. aeruginosa* was 64 μg ml^−1^. Acetone and methanol extracts *of Rumex obtusifolius* had 128 μg ml^−1^ MIC value *against B. subtilis* and *P. aeruginosa.* On the other hand, selected plants’ crude extracts possessed broad range of activity inhibiting the growth of Gram-negative, Gram-positive and endospore forming bacteria. They also showed considerable activity against yeasts. Among the tested five plants *Agrimonia eupatoria*, *Rumex obtusifolius* and *Sanguisorba officinalis* had lower MIC values against yeast strains, compared to *Hypericum alpestre* and *Lilium armenum,* which in contrast, inhibited the growth of yeast strains only in high concentrations.

**Table 3:** Determination of minimum inhibitory concentration (MIC) and Minimum bactericidal/fungicidal concentrations (MBC/MFC) of crude extracts of five selected plant materials. (Place in the text: page 10 after line 13)

Plant species	Extract^a^	Test strains^b^/ MIC (μg ml^−1^) and MBC/MFC(μg ml^−1^)													
		*SA*		*BS*		*PA*		*EC*		*ST*		*CG*		*CA*	
		MIC	MBC	MIC	MBC	MIC	MBC	MIC	MBC	MIC	MBC	MIC	MBC	MIC	MBC
*Hypericum alpestre* (aerial part)	Wat	1024	–	–	–	256	–	–	–	1024		–	–	–	–
	Met	256	512	256	512	128	1024	512	–	–	–	–	–	–	–
	CF	128	–	256	1024	256	1024	1024	–	1024	–	1024	–	–	–
	Ace	128	–	128	512	64	512	1024	–	512	–	1024	–	1024	–
	Hex	128	512	256	512	64	512	1024	–	1024	–	1024	–	–	–
*Agrimonia eupatoria* (whole plant)	Wat	1024	–	–	–	1024	–	1024	–	1024	–	1024	–	–	–
	Met	256	–	128	–	256	1024	512	–	1024	–	1024	–	–	–
	CF	256	512	256	1024	512	1024	512	–	512	–	256	1024	512	–
	Ace	256	–	128	1024	128	512	512	–	512	–	256	–	512	–
	Hex	256	1024	256	1024	128	512	–	–	512	–	512	1024	1024	–
*Lilium armenum* (bulb)	Wat	1024	–	1024	–	1024	–	–	–	1024	–	–	–	–	–
	Met	512	–	512	1024	256	512	1024	–	–	–	1024	–	–	–
	CF	512	–	1024	–	128	512	1024	–	512	–	512	1024	1024	–
	Ace	512	–	512	–	128	512	512	–	512	–	–	–	–	–
	Hex	1024	–	1024	–	256	–	–	–	1024	–	–	–	–	–
*Rumex obtusifolius* (seed)	Wat	512	–	1024	–	256	1024	–	–	1024		1024	–	1024	–
	Met	256	–	128	–	128	512	512	–	512		512	–	–	–
	CF	1024	–	1024	–	512	–	–	–	1024	–	1024	–	–	–
	Ace	256	–	128	–	128	1024	512	–	512		512	–	–	–
	Hex	512	–	512	–	512	1024	–	–	1024	–	1024	–	1024	–
*Sanguisorba officinalis* (aerial part)	Wat	–	–	1024	–	512	–	–	–	–	–	–	–	–	–
	Met	256	–	128	512	256	512	512	–	512	–	512	–	512	–
	CF	512	–	512	–	128	256	1024		1024		512	1024	512	
	Ace	128	–	128	1024	64	256	256	–	256	–	512	–	512	–
	Hex	256	–	512	1024	256	512	1024	–	1024	–	256	–	512	–
	PC^d^	0.25	0.5	0.25	0.5	0.25	0.5	0.5	2	1	>2	2	>4	2	4

^a^ Extracts: Wat- Water, Met –Methanol, CF-Chloroform, Ace– Acetone, Hex - Hexane

^b^ Test strains: *Escherichia coli* WKPM-M17 (EC), *Pseudomonas aeruginosa* GRP3 (VKPH B-82–5) (PA), *Bacillus subtilis* WT-A1 (BS)*, Salmonella typhimurium* WDCM 1754 (ST)*, Staphylococcus aureus* WDCM 5233 (SA), *Candida albicans* 174 (CA), *Candida guilliermondii* HP-17 (CG)

^c^ (–): MIC/MBC/MFC value was >1024 μg ml^−1^

^d^ PC: positive control (gentamicin (range 2–0.003 μg ml^−1^) (for bacteria), nystatin (range 4–0.125 μg ml^−1^) (for yeasts))

All experiments were repeated three times and average means were represented

Comparison of obtained MIC values of the tested plant materials with literature data showed both similaraities and differences. Wegiera et al. [27] showed that ethanol extracts of *Rumex confertus* seeds had 62.5–125 μg ml^−1^ MIC values against *S. aureus* strains which is in coincidence with our data. The results were also similar in case of *E. coli*. However, in case of *P. aeruginosa* and *C. albicans* the MIC values obtained by Wegiera et al. [27] were different from our results. In our experiments methanol and acetone extracts of *Rumex obtusifolius* had 128 μg ml^−1^ MIC value against *P. aeruginosa*, whereas its MIC was above the 500 μg ml^−1^ in the other research [27]. The MIC against *C. albicans* were above 1024 μg ml^−1^ in our experiments. In contrast, it was 250 μg ml^−1^ in research done by Wegiera et al. [27]. These differences could be explained by differential susceptibility of the used test strains. Copland et al. [28] in their research showed that only hexane extracts of *Agrimonia eupatoria* seeds (they tested methanol and dichloromethane extracts as well) showed activity against *B. subtilis* with 750 μg ml^−1^ MIC value at tested concentrations. They did not find antimicrobial activity against tested *P. aeruginosa* and *S. aureus* strains. In contrast, Watkins et al. [29] who used water, red wine, as well as 25% and 75% ethanol as solvents, showed that *Agrimonia eupatoria* had moderate antimicrobial activity against *S. aureus, B. subtilis* and *E. coli.* Antimicrobial activity of ethanol extract of *Agrimonia eupatoria* against *Helicobacter pylori* was also shown [30]. Kokoska et al. [25] showed that ethanol extracts of *Sanguisorba officinalis* had 62.5–250 μg ml^−1^ MIC values against *E. coli, S. aureus and P. aeruginosa* which partially coincides with our data. There was no evidence in literature about antimicrobial activity of *Lilium armenum* and *Hypericum alpestre*.

### Determination of MBC/MFC values of the selected plant materials

MBC/MFC of selected five plant materials was determined. Three of the tested five plant species *Agrimonia eupatoria, Sanguisorba officinalis* and *Lilium armenum* possessed fungicidal activity against *C. guilliermondii* (see Table 3)*.* Only *Hypericum alpestre* and *Agrimonia eupatoria* extracts killed *S. aureus* cells. In contrast, all five plant materials expressed bactericidal activity against *P. aeruginosa:* some of them even at 256 μg ml^−1^ concentrations (chloroform and acetone extracts of *Sanguisorba officinalis*). Within tested five plant species only *Rumex obtusifolius* had no bactericidal activity against *B. subtilis* at tested concentrations. This is particularly interesting as tested *B. subtilis* strain could have high resistance according to our previous work [17]. Cidal activity of almost all tested plant materials toward this endospore forming bacteria also allowed us to assume that they possessed activity against endospores as well. All tested plant materials showed only static activity against *E. coli, S. typhimurium and C. albicans* till the concentration of 1024 μg ml^−1^. The obtained data indicated that MBC/MFC concentrations were two-three times higher than respective MIC values.

We did not find any research about bactericidal/fungicidal activity of all tested five plant species*.* Therefore, bactericidal/fungicidal activity of these plants was investigated for the first time.

### Evaluation of tested solvents’ efficiency

Since we used five solvents in our screening protocol, the collected data allowed us to evaluate solvents for their efficiency to solubilize antimicrobial compounds from plant materials as well as their other properties. The obtained results (see Tables 2 and 3) showed that acetone extracts demonstrated the highest antimicrobial activities for almost all tested plant materials. Methanol extracts showed the second strongest antimicrobial activity, followed by chloroform, hexane and water. Methanol was the best solvent in regard of quantity of extracted materials, followed by water, acetone, chloroform and hexane. During extraction with hexane, only small amount of dry material was harvested. From the point of easy handling (quick evaporation, easy harvesting and weighing, high further solubility in DMSO, absence of residual moisture) we considered methanol as the best solvent, followed by acetone, water, chloroform and hexane. Our considerations about solvent efficiency agree with literature data. According to Eloff [9], acetone received the best overall rating for yielding antimicrobials from plant materials among the tested six common solvents (methylene dichloride, methanol, ethanol, water, acetone, and a mixture of chloroform, methanol and water). However, Eloff [9] conducted his study only on two plant materials, therefore, there was a necessity for more data for generalization of effectiveness of acetone as an extractant. Mothana and Lindequist [31] showed that methanol extracts were more active than chloroform extracts which confirms our data. In our study aqueous extracts have shown poor antimicrobial activities at tested concentrations which coincides with literature data [3, 10, 31–33].

## Conclusions

Thus, antimicrobial activity of five plant materials was evaluated against five bacterial and two yeast strains. Some of the plants had quite low MIC/MBC and therefore have prospective for further more comprehensive studies. We also established that acetone was the most effective solvent among the tested five solvents for solubilizing antimicrobial compounds from the tested plant materials. Hence, we propose using acetone in antimicrobial screening protocols. In further research we will to try to purify the plants’ active compounds responsible for their antimicrobial action. Synergistic effect of these five plants extracts with commonly used antibiotics is also interesting.

## Additional file

Additional file 1:
**Table S1.** The list of initially tested 28 wild plant species with common names, family names, tested parts and traditional uses in Armenian folk medicine. **Table S2.** Antibacterial and anti-yeast activity of crude extracts of tested 28 wild plant species determined by agar well diffusion assay. (DOCX 47 kb)

## Abbreviations

Ace: Acetone
BS: *Bacillus subtilis*
CA: *Candida albicans*
CF: Chloroform
CG: *Candida guilliermondii*
EC: *Escherichia coli*
Hex: Hexane
Met: Methanol
PA: *Pseudomonas aeruginosa*
PC: Positive control
SA: *Staphylococcus aureus*
ST: *Salmonella typhimurium*
Wat: Water.
