# Supplementary material for: Antimicrobial activity of some plant materials used in Armenian traditional medicine
Source: BMC Complement Altern Med. 2017 Jan 17;17:50. doi: 10.1186/s12906-017-1573-y (PMC5240328; doi:10.1186/s12906-017-1573-y)
Supplement: Additional file 1: — Table S1. The list of initially tested 28 wild plant species with common names, family names, tested parts and traditional uses in Armenian folk medicine. Table S2. Antibacterial and anti-yeast activity of crude extracts of tested 28 wild plant species determined by agar well diffusion assay. (DOCX 47 kb) [file 12906_2017_1573_MOESM1_ESM.docx]

# Additional file for manuscript “Antimicrobial activity of some plant materials used in Armenian folk medicine”.

**Table 1. The list of initially tested 28 wild plant species with common names, family names, tested parts and traditional uses in Armenian folk medicine.**

| **Plant name^a^** | **Common name** | **Family** | **Part tested** | **Traditional uses^b^** |
| --- | --- | --- | --- | --- |
| *Achillea filipendulina* Lam. | yarrow | Compositae | aerial part | Purulent wounds, oral, colon and other inflammations |
| *Achillea nobilis*subsp*.  neilreichii*(A.Kern.) Takht. | yarrow | Compositae | aerial part | Purulent wounds, oral, colon and other inflammations |
| *Agrimonia eupatoria* L. | common agrimony | Rosaceae | aerial part | Hepatitis, nephritis, stomatitis, yellow fever, purulent wounds |
| *Alchemilla sericata* Rchb. ex Buser | lady's mantle | Rosaceae | aerial part | Purulent wounds, eyelid inflammations |
| *Alchemilla vulgaris* Willd. | lady's mantle | Rosaceae | aerial part | Purulent wounds, eyelid inflammations |
| *Chelidonium majus* L. | greater celandine | Papaveraceae | whole plant | Various skin conditions (wart, scabies, cutaneous tuberculosis), wounds during venereal diseases, purulent wounds, gastrointestinal inflammations, dysentery, syphilis, malaria, helminthiasis, fungal diseases *etc.* |
| *Cichorium intybus* L. | common chicory | Asteraceae | aerial part,  root | Hepatitis, nephritis, cholecystitis, mucosal inflammations, malaria, fungal infections |
| *Cuscuta europaea* L. | greater dodder | Convolvulaceae | whole plant | Helminthiasis, malaria quartana, cholecystitis, gastritis, splenomegaly, hepatitis |
| *Gentiana cruciata* L. | star gentian | Gentianaceae | aerial part,  root | Mucosal inflammation, hepatitis, splenomegaly |
| *Hypericum alpestre* subsp. *polygonifolium* (Rupr.) Avet. & Takht. | hypericum | [H](https://en.wikipedia.org/wiki/Hypericaceae)ypericaceae | aerial part | Pneumonia, wounds, hepatitis, cholecystitis gastrointestinal inflammation, nephritis, skin diseases |
| *Inula helenium* L. | horse-heal | Asteraceae | leaf, flower | Gastrointestinal inflammation, whooping cough, yellow fever |
| *Leonurus cardiaca* L. | motherwort | Lamiaceae | aerial part | Cardiac muscle inflammation |
| *Lilium armenum* (Miscz. ex Grossh.) Manden. | unknown | [Liliumceae](https://en.wikipedia.org/wiki/Liliaceae) | leaf with stalk,  bulb | Whooping cough, purulent wounds, burns, leprosy, fungal diseases, mastitis, cystitis |
| *Origanum vulgare* L. | wild marjoram | Lamiaceae | aerial part | Gastritis, whooping cough, yellow fever |
| *Polygonatum odoratum* (Mill.) Druce | angular Solomon's seal | Asparagaceae | rhizome, aerial part | Lymphnode inflammation, abscesses |
| *Peganum harmala* L. | wild rue | Nitrariaceae | seed, root, stalk with leaf, flower | Gastritis, intestinal inflammation, nephritis, leprosy |
| *Rubus anatolicus* Focke | holy bramble | Rosaceae | leaf with flower | Leprosy, gastrointestinal, inflammation, hepatitis, nephritis, yellow fever, burns |
| *Rumex obtusifolius* L. | asiatic dock | Polygonaceae | leaf, root, inflorescence seed | Infectious diseases, skin rash, mucosal inflammation |
| *Sambucus ebulus* L. | danewort | Adoxaceae | leaf, inflorescence fruit | Various inflammations |
| *Sambucus nigra* L. | elderberry | Adoxaceae | leaf, inflorescence fruit | Bronchitis, stomatitis, tonsillitis, dysentery, erysipelas, various inflammations |
| *Sanguisorba officinalis* L. | great burnet | Rosaceae | aerial part, root | Tonsillitis, purulent wounds, inflammations, skin infections, dysentery, typhoid fever, trichomoniasis, stomatitis, gingivitis, flu |
| *Stachys sylvatica* L. | hedge woundwort | Lamiaceae | aerial part | Wounds |
| *Thymus serpyllum* L. | wild thyme | Lamiaceae | aerial part | Otitis, gastrointestinal inflammation, hepatitis, nephritis, yellow fever, leprosy |
| *Tilia caucasica* Rupr. | basswood | Malvaceae | leaf, inflorescence | Tonsillitis, stomatitis, red measles, mumps, flu, various inflammations, pneumonia *etc.* |
| *Veratrum album* L. | European white hellebore | Melanthiaceae | aerial part,  root | Tuberculosis, pleurisy, purulent wounds, arthritis, hepatitis, pneumonia |
| *Verbascum thapsus* L. | common mullein | Scrophulariaceae | leaf, inflorescence root | Whooping cough, cough, hepatitis |
| *Veronica anagallis-aquatica* L. | water speedwell | [Plantaginaceae](https://en.wikipedia.org/wiki/Plantaginaceae) | aerial part | Burns |
| *Viscum album* L. | common mistletoe | Santalaceae | whole plant | Inflammation of lymphnode and other organs, diarrhea helminthiasis, abscesses, wounds, tuberculosis |

^a^ The plants names has been checked with <http://www.theplantlist.org/>

^b^ In this section there are presented only such traditional plants which can imply the presence of antimicrobial compound.

**Table 2. Antibacterial and anti-yeast activity of crude extracts of tested 28 wild plant species determined by agar well diffusion assay.**

| **Plant species** | **Part tested** | **Extract** | **Diameter of growth inhibition zone with standard deviation (mm)** | | | | | | |
| --- | --- | --- | --- | --- | --- | --- | --- | --- | --- |
|  |  |  | ***SA^a^*** | ***BS*** | ***PA*** | ***EC*** | ***ST*** | ***CG*** | ***CA*** |
| *Achillea filipendulina* | leaf | Water | – | – | – | – | – | – | – |
|  |  | Methanol | – | – | – | – | 9±0.6 | – | – |
|  |  | Chloroform | – | – | – | – | – | – | – |
|  |  | Acetone | – | – | – | – | – | – | – |
|  |  | Hexane | – | – | – | – | – | – | – |
| *Achillea nobilis* | aerial part | Water | – | – | – | – | – | – | – |
|  |  | Methanol | – | – | 9±0.6 | – | – | – | – |
|  |  | Chloroform | – | – | 9±0.6 | – | – | – | – |
|  |  | Acetone | 9±0.6 | – | 11±0.6 | – | – | – | – |
|  |  | Hexane | 9±0.6 | – | 10±0.6 | – | – | – | – |
| *Agrimonia eupatoria* | whole plant | Water | – | – | – | 9±0.6 | – | – | – |
|  |  | Methanol | 10±0.6 | 11±0.6 | 11±0.6 | 11±0.6 | – | 11±0.6 | – |
|  |  | Chloroform | 9 | 11±0.6 | 10±0.6 | 10±0.6 | 11±0.6 | 12±0.6 | 9±0.6 |
|  |  | Acetone | 12±0.6 | 10±0.6 | 11±0.6 | 10±0.6 | 10±0.6 | 12±1 | 9±0.6 |
|  |  | Hexane | 13±0.6 | 10±0.6 | 10±0.6 | – | 9±0.6 | 9±0.6 | – |
| *Alchemilla sericata* | aerial part | Water | – | – | – | 9±0.6 | – | – | – |
|  |  | Methanol | 10±0.6 | 9±0.6 | 10±0.6 | 9±0.6 | – | 11±0.6 | – |
|  |  | Chloroform | – | – | 9±0.6 | 9±0.6 | – | 10±0.6 | – |
|  |  | Acetone | – | – | 10±0.6 | – | – | – | – |
|  |  | Hexane | – | – | 9±0.6 | – | – | – | – |
| *Alchemilla vulgaris* | whole plant | Water | – | – | 10±0.6 | – | – | 9±0.6 | 9±0.6 |
|  |  | Methanol | – | 9±0.6 | 9±0.6 | 9±0.6 | – | 9±0.6 | – |
|  |  | Chloroform | – | – | 9±0.6 | 9±0.6 | – | 10±0.6 | – |
|  |  | Acetone | – | 10±0.6 | – | 11±0.6 | – | – | 10±0.6 |
|  |  | Hexane | – | 9±0.6 | – | 9±0.6 | – | – | – |
| *Chelidonium majus* | aerial part | Water | – | – | – | – | – | – | – |
|  |  | Methanol | – | 9±0.6 | 10±0.6 | – | – | – | – |
|  |  | Chloroform | – | – | 10±0.6 | – | – | – | – |
|  |  | Acetone | – | – | 9±0.6 | – | – | 9±0.6 | – |
|  |  | Hexane | – | – | 9±0.6 | – | – | – | – |
| *Cichorium intybus* | aerial part | Water | 9±0.6 | – | – | – | 9±0.6 | 9±0.6 | – |
|  |  | Methanol | 10±0.6 | – | – | – | 10±0.6 | – | – |
|  |  | Chloroform | 11±0.6 | – | – | 9±0.6 | 11±0.6 | 10±1 | – |
|  |  | Acetone | 10±0.6 | – | – | 10±0.6 | 10±0.6 | 10±0.6 | – |
|  |  | Hexane | 10±0.6 | – | – | – | – | – | – |
| *Cichorium intybus* | root | Water | 9±0.6 | 9±0.6 | – | – | – | – | – |
|  |  | Methanol | 10±0.6 | 9±0.6 | – | – | – | – | – |
|  |  | Chloroform | – | – | 10±0.6 | – | – | – | – |
|  |  | Acetone | 10±0.6 | 10±0.6 | 10±0.6 | – | – | – | – |
|  |  | Hexane | – | – | – | – | – | – | – |
| *Cuscuta europaea* | whole plant | Water | – | – | – | – | – | – | – |
|  |  | Methanol | – | – | 11±0.6 | 9±0.6 | – | – | – |
|  |  | Chloroform | – | – | 10±0.6 | 9±0.6 | – | 9±0.6 | – |
|  |  | Acetone | – | – | 12±0.6 | – | – | 9±0.6 | – |
|  |  | Hexane | 9±0.6 | – | 14±0.6 | 9±0.6 | 9±0.6 | 9±0.6 | – |
| *Gentiana crusiata* | root | Water | – | – | 12±0.6 | – | – | – | – |
|  |  | Methanol | – | – | 12±0.6 | – | – | – | – |
|  |  | Chloroform | – | – | 9±0.6 | – | – | – | – |
|  |  | Acetone | – | – | 10±0.6 | – | – | – | – |
|  |  | Hexane | – | – | – | – | – | – | – |
| *Gentiana crusiata* | aerial part | Water | – | – | 12±0.6 | 10±0.6 | – | – | 11±0.6 |
|  |  | Methanol | – | – | 11±0.6 | – | – | – | 9±0.6 |
|  |  | Chloroform | – | – | – | – | – | – | – |
|  |  | Acetone | – | – | – | – | – | – | – |
|  |  | Hexane | – | – | – | – | – | – | – |
| *Gentiana crusiata* | root | Water | – | – | 12±0.6 | – | – | – | – |
|  |  | Methanol | – | – | 12±0.6 | – | – | – | – |
|  |  | Chloroform | – | – | 9±0.6 | – | – | – | – |
|  |  | Acetone | – | – | 10±0.6 | – | – | – | – |
|  |  | Hexane | – | – | – | – | – | – | – |
| *Hypericum alpestre* | aerial part | Water | – | – | 11±0.6 | – | – | – | – |
|  |  | Methanol | 10±0.6 | 11±0.6 | 18±0.6 | 10±0.6 | – | – | – |
|  |  | Chloroform | 13±0.6 | 12±0.6 | 23±1 | – | – | – | – |
|  |  | Acetone | 15±0.6 | 13±0.6 | 21±0.6 | – | 10±0.6 | 9±0.6 | – |
|  |  | Hexane | 17±0.6 | 16±0.6 | 21±0.6 | – | – | – | – |
| *Inula helenium* | leaf | Water | – | – | – | – | – | – | – |
|  |  | Methanol | – | – | 9±0.6 | – | – | – | – |
|  |  | Chloroform | – | – | 9±0.6 | – | – | – | – |
|  |  | Acetone | – | – | 9±0.6 | – | – | – | – |
|  |  | Hexane | – | – | – | – | – | – | – |
| *Inula helenium* | flower | Water | – | – | – | – | – | – | – |
|  |  | Methanol | – | – | 10±0.6 | – | – | – | – |
|  |  | Chloroform | – | – | – | – | – | – | – |
|  |  | Acetone | – | – | – | – | 9±0.6 | – | – |
|  |  | Hexane | – | – | – | – | 9±0.6 | – | – |
| *Leonurus cardiaca* | aerial part | Water | – | – | – | – | – | – | – |
|  |  | Methanol | – | – | – | – | – | – | – |
|  |  | Chloroform | 9±0.6 | – | – | – | – | – | – |
|  |  | Acetone | 9±0.6 | – | – | – | – | – | – |
|  |  | Hexane | – | – | – | – | – | – | – |
| *Lilium armenum* | aerial part | Water | – | – | 10±0.6 | – | – | – | – |
|  |  | Methanol | 9±0.6 | – | 13±0.6 | – | – | – | – |
|  |  | Chloroform | 9±0.6 | – | 10±0.6 | – | – | – | – |
|  |  | Acetone | 10±0.6 | 9 | 11±0.6 | – | – | – | – |
|  |  | Hexane | 9±0.6 | – | 12±0.6 | – | – | – | – |
| *Lilium armenum* | bulb | Water | – | – | 9±0.6 | – | – | – | – |
|  |  | Methanol | 9±0.6 | 9±0.6 | 11±0.6 | 9±0.6 | – | – | – |
|  |  | Chloroform | 9±0.6 | – | 13±0.6 | – | 9±0.6 | – | – |
|  |  | Acetone | 10±0.6 | 9±0.6 | 11±0.6 | 9±0.6 | 9±0.6 | – | – |
|  |  | Hexane | – | – | 11±0.6 | – | 9±0.6 | – | – |
| *Origanum vulgare* | aerial part | Water | – | – | – | – | – | – | – |
|  |  | Methanol | 9±0.6 | – | – | – | 9±0.6 | 9±0.6 | 10±0.6 |
|  |  | Chloroform | 9±0.6 | – | – | – | 9±0.6 | – | 9±0.6 |
|  |  | Acetone | – | – | – | – | – | – | – |
|  |  | Hexane | – | – | – | – | – | – | – |
| *Polygonatum odoratum* | rhizome | Water | – | – | – | – | – | – | – |
|  |  | Methanol | – | – | 10±0.6 | 9±0.6 | – | – | – |
|  |  | Chloroform | – | – | 11±0.6 | 10±0.6 | – | – | – |
|  |  | Acetone | – | – | 11±0.6 | 10±0.6 | 10±0.6 | 10±0.6 | – |
|  |  | Hexane | – | – | 9±0.6 | – | – | – | – |
| *Polygonatum odoratum* | aerial part | Water | – | – | – | – | – | – | – |
|  |  | Methanol | 9 | – | – | 9±0.6 | – | – | – |
|  |  | Chloroform | 9±0.6 | – | 11±0.6 | – | – | – | – |
|  |  | Acetone | 9±0.6 | – | 10±0.6 | 9±0.6 | – | – | – |
|  |  | Hexane | 9±0.6 | – | 10±0.6 | 9±0.6 | – | – | – |
| *Peganum harmala* | seed | Water | – | – | 10±0.6 | – | 10±0.6 | – | – |
|  |  | Methanol | – | – | 11±0.6 | – | 10±0.6 | – | – |
|  |  | Chloroform | – | – | 10±0.6 | – | – | 11±0.6 | – |
|  |  | Acetone | – | – | 10±0.6 | – | 10±0.6 | 10±0.6 | – |
|  |  | Hexane | – | – | 10±0.6 | – | 10±0.6 | – | – |
| *Peganum harmala* | root | Water | – | – | – | – | – | – | – |
|  |  | Methanol | – | – | – | – | – | – | – |
|  |  | Chloroform | – | – | – | 9±0.6 | – | – | – |
|  |  | Acetone | – | – | 9±0.6 | 9±0.6 | – | – | – |
|  |  | Hexane | – | – | 9±0.6 | 10±0.6 | – | – | – |
| *Peganum harmala* | bark with leaf | Water | – | – | 10±0.6 | – | – | – | – |
|  |  | Methanol | – | – | 9±0.6 | – | – | – | – |
|  |  | Chloroform | – | – | – | 9±0.6 | – | – | – |
|  |  | Acetone | – | – | – | 11±0.6 | 10±0.6 | – | – |
|  |  | Hexane | – | – | – | – | 9±0.6 | – | – |
| *Peganum harmala* | flower | Water | – | – | – | – | – | – | – |
|  |  | Methanol | – | – | – | 9±0.6 | – | – | – |
|  |  | Chloroform | – | – | – | 9±0.6 | – | – | – |
|  |  | Acetone | – | 9±0.6 | 9±0.6 | 9±0.6 | 9±0.6 | – | – |
|  |  | Hexane | – | 9±0.6 | 13±0.6 | – | – | – | – |
| *Rubus anatolicus* | leaves with flowers | Water | – | – | 9±0.6 | – | – | – | – |
|  |  | Methanol | – | – | 12±0.6 | – | 9±0.6 | – | 9±0.6 |
|  |  | Chloroform | – | – | 12±0.6 | – | 9±0.6 | 14±0.6 | – |
|  |  | Acetone | – | – | 11±0.6 | – | 9±0.6 | 10±0.6 | 9±0.6 |
|  |  | Hexane | 10±0.6 | – | – | – | – | 9±0.6 | 9±0.6 |
| *Rumex obtusifolius* | leaf | Water | – | – | – | – | – | – | – |
|  |  | Methanol | 9±0.6 | – | – | – | 10±0.6 | 14±0.6 | – |
|  |  | Chloroform | 11±0.6 | – | 10±0.6 | – | 10±0.6 | 9±0.6 | – |
|  |  | Acetone | 11±0.6 | – | 12±0.6 | – | 10±0.6 | 12±0.6 | – |
|  |  | Hexane | 9±0.6 | – | 11±0.6 | 9±0.6 | – | – | – |
| *Rumex obtusifolius* | root | Water | – | – | 11±0.6 | – | – | – | – |
|  |  | Methanol | 10±0.6 | – | 10±0.6 | – | – | – | 9±0.6 |
|  |  | Chloroform | 11±0.6 | – | 10±0.6 | – | 9±0.6 | 12±0.6 | 9±0.6 |
|  |  | Acetone | 10±0.6 | 9±0.6 | 12±0.6 | – | – | 13±0.6 | 9±0.6 |
|  |  | Hexane | 9±0.6 | – | – | – | – | 12±0.6 | – |
| *Rumex obtusifolius* | inflorescence | Water | – | – | – | – | – | – | – |
|  |  | Methanol | – | 9±0.6 | 9±0.6 | 10±0.6 | – | – | – |
|  |  | Chloroform | – | 9±0.6 | 9±0.6 | 9±0.6 | – | – | – |
|  |  | Acetone | 11±0.6 | 11±0.6 | 9±0.6 | 10±0.6 | 9±0.6 | 9±0.6 | – |
|  |  | Hexane | 9±0.6 | 9±0.6 | 10±0.6 | – | – | – | – |
| *Rumex obtusifolius* | seed | Water | 9±0.6 | – | 10±0.6 | – | – | – | – |
|  |  | Methanol | 12±0.6 | 11±0.6 | 10±0.6 | 11±0.6 | 12±0.6 | 10±0.6 | – |
|  |  | Chloroform | – | 9±0.6 | 9±0.6 | – | – | – | – |
|  |  | Acetone | 12±0.6 | 12±0.6 | 10±0.6 | 10±0.6 | 12±0.6 | 10±0.6 | – |
|  |  | Hexane | 9±0.6 | 9±0.6 | 10±0.6 | – | – | – | – |
| *Sambucus ebulus* | leaf | Water | – | – | – | – | – | – | – |
|  |  | Methanol | – | – | – | 9±0.6 | – | – | – |
|  |  | Chloroform | – | – | – | 9±0.6 | – | – | – |
|  |  | Acetone | – | – | – | 9±0.6 | – | – | – |
|  |  | Hexane | – | – | – | – | – | – | – |
| *Sambucus ebulus* | inflorescence | Water | 9±0.6 | – | 11±0.6 | – | 10±0.6 | – |  |
|  |  | Methanol | 9±0.6 | – | 12±0.6 | 9±0.6 | – | 11±0.6 | – |
|  |  | Chloroform | – | 9±0.6 | 12±0.6 | – | – | – | – |
|  |  | Acetone | – | – | 12±0.6 | – | – | – | – |
|  |  | Hexane | – | – | – | – | 12±0.6 | 9±0.6 | – |
| *Sambucus ebulus* | fruit | Water | – | – | – | – | 9±0.6 | – | – |
|  |  | Methanol | – | – | – | – | 9±0.6 | – | – |
|  |  | Chloroform | – | 9±0.6 | – | – | – | – | – |
|  |  | Acetone | – | 11±0.6 | 9±0.6 | – | – | – | – |
|  |  | Hexane | – | 9±0.6 | 9±0.6 | – | – | – | – |
| *Sambucus nigra* | leaf | Water | 9±0.6 | – | – | 11±0.6 | 9±0.6 | 10±1 | – |
|  |  | Methanol | 11±0.6 | 9±0.6 | – | 11±0.6 | – | 11±0.6 | – |
|  |  | Chloroform | 10±0.6 | – | – | 10±0.6 | – | 10±0.6 | – |
|  |  | Acetone | – | – | – | – | – | 9±0.6 | – |
|  |  | Hexane | 9±0.6 | – | – | – | 9±0.6 | – | – |
| *Sambucus nigra* | inflorescence | Water | – | – | – | – | – | – | – |
|  |  | Methanol | – | – | 9±0.6 | – | – | – | – |
|  |  | Chloroform | – | – | 11±0.6 | – | – | – | – |
|  |  | Acetone | – | 9±0.6 | 11±0.6 | – | – | 9±0.6 | – |
|  |  | Hexane | – | – | 9±0.6 | – | – | 9±0.6 | – |
| *Sambucus nigra* | fruit | Water | 9±0.6 | – | 9±0.6 | – | – | – | – |
|  |  | Methanol | 9±0.6 | – | 10±0.6 | – | – | – | – |
|  |  | Chloroform | 10±0.6 | – | 10±0.6 | – | – | – | – |
|  |  | Acetone | 11±0.6 | 9±0.6 | 10±0.6 | – | – | – | – |
|  |  | Hexane | 9±0.6 | 9±0.6 | 9±0.6 | – | – | – | – |
| *Sanguisorba officinalis* | aerial part | Water | – | – | 10±0.6 | – | 9±0.6 | – | – |
|  |  | Methanol | 12±0.6 | 11±0.6 | 10±0.6 | 10±0.6 | 12±0.6 | 9±0.6 | 10±0.6 |
|  |  | Chloroform | 10±0.6 | 9±0.6 | 10±0.6 | – | – | 12±0.6 | 11±0.6 |
|  |  | Acetone | 13±0.6 | 13±0.6 | 12±1 | 12±0.6 | 11±0.6 | 10±0.6 | 10±0.6 |
|  |  | Hexane | 11±0.6 | 10±0.6 | 10±0.6 | 9±0.6 | – | 10±0.6 | 9±0.6 |
| *Sanguisorba officinalis* | root | Water | – | – | – | – | – | – | – |
|  |  | Methanol | 10±0.6 | – | 10±0.6 | 10±0.6 | – | 10±0.6 | 10±0.6 |
|  |  | Chloroform | – | – | – | – | – | 10±0.6 | 11±0.6 |
|  |  | Acetone | 10±0.6 | 10±0.6 | 10±0.6 | 10±0.6 | – | 10±0.6 | 10±0.6 |
|  |  | Hexane | – | – | – | – | – | – | 10±0.6 |
| *Stachys sylvatica* | aerial part | Water | – | – | 10±0.6 | – | – | – | – |
|  |  | Methanol | – | – | 12±0.6 | – | – | – | – |
|  |  | Chloroform | – | 10±0.6 | 11±0.6 | – | – | – | – |
|  |  | Acetone | – | 9±0.6 | 11±0.6 | – | – | – | – |
|  |  | Hexane | – | – | 10±0.6 | – | – | – | – |
| *Thymus serpyllum* | aerial part | Water | – | – | – | – | – | – | – |
|  |  | Methanol | 9±0.6 | – | – | – | – | – | – |
|  |  | Chloroform | – | – | 9±0.6 | 10±0.6 | – | – | – |
|  |  | Acetone | – | – | – | 9±0.6 | – | – | – |
|  |  | Hexane | 10±0.6 | – | – | 9±0.6 | – | – | – |
| *Tilia caucasica* | leaf | Water | – | – | – | – | – | – | – |
|  |  | Methanol | – | – | 9±0.6 | – | – | – | – |
|  |  | Chloroform | – | – | – | 9±0.6 | 9±0.6 | 9±0.6 | – |
|  |  | Acetone | – | – | – | 10±0.6 | 9±0.6 | 9±0.6 | – |
|  |  | Hexane | – | – | – | 9±0.6 | – | – | – |
| *Tilia caucasica* | flower | Water | – | – | – | – | – | – | – |
|  |  | Methanol | – | – | 10±0.6 | – | 9±0.6 | – | – |
|  |  | Chloroform | – | – | – | – | 9±0.6 | – | – |
|  |  | Acetone | 9±0.6 | 9±0.6 | 10±0.6 | – | 9±0.6 | 9±0.6 | – |
|  |  | Hexane | – | – | 9±0.6 | – | – | 9±0.6 | – |
| *Veratrum album* | aerial part | Water | – | – | 13±0.6 | – | – | 12±0.6 | – |
|  |  | Methanol | – | – | 12±0.6 | – | – | – | – |
|  |  | Chloroform | – | – | 11±0.6 | 9±0.6 | – | – | – |
|  |  | Acetone | 9±0.6 | – | 11±0.6 | 9±0.6 | – | 10±0.6 | – |
|  |  | Hexane | 9±0.6 | – | 10±0.6 | – | – | – | – |
| *Veratrum album* | root | Water | 10±0.6 | – | – | – | – | 12±0.6 | – |
|  |  | Methanol | – | – | – | 10±0.6 | 10±0.6 | – | – |
|  |  | Chloroform | – | – | – | – | – | – | – |
|  |  | Acetone | 10±0.6 | – | – | – | 10±0.6 | 10±0.6 | – |
|  |  | Hexane | – | – | – | – | – | 11±0.6 | – |
| *Verbascum thapsus* | leaf | Water | – | 9±0.6 | – | – | – | – | – |
|  |  | Methanol | – | – | – | – | – | – | – |
|  |  | Chloroform | – | 9±0.6 | – | – | – | – | – |
|  |  | Acetone | – | – | – | – | – | – | – |
|  |  | Hexane | – | – | – | – | – | – | – |
| *Verbascum thapsus* | inflorescence | Water | – | – | – | – | – | – | – |
|  |  | Methanol | – | – | 9±0.6 | 9±0.6 | – | – | – |
|  |  | Chloroform | 9±0.6 | – | – | 10±0.6 | – | – | – |
|  |  | Acetone | 9±0.6 | 9±0.6 | 10±0.6 | 10±0.6 | 10±0.6 | – | – |
|  |  | Hexane | – | – |  | – | – | – | – |
| *Verbascum thapsus* | root | Water | – | – | – | – | – | – | – |
|  |  | Methanol | – | – | 9±0.6 | – | – | – | – |
|  |  | Chloroform | – | – | 10±0.6 | – | – | – | – |
|  |  | Acetone | – | – | 10±0.6 | – | – | – | – |
|  |  | Hexane | – | – | – | – | – | – | – |
| *Veronica anagallis* | aerial part | Water | – | – | – | – | – | – | – |
|  |  | Methanol | 10±0.6 | – | – | – | – | 10±1 | – |
|  |  | Chloroform | – | – | – | – | – | – | – |
|  |  | Acetone | – | – | – | – | – | – | – |
|  |  | Hexane | 9 | – | 9±0.6 | – | – | – | – |
| *Viscum album* | whole plant | Water | – | – | 10±0.6 | – | – | – | – |
|  |  | Methanol | – | 9±0.6 | – | – | – | – | – |
|  |  | Chloroform | 10±0.6 | – | – | 9±0.6 | – | – | – |
|  |  | Acetone | 9±0.6 | – | 9±0.6 | 9±0.6 | – | – | – |
|  |  | Hexane | – | – | – | – | – | – | – |
|  | **PC^b^** |  | 20±0.6 | 30±1 | 28±1 | 19±0.6 | 23±0.6 | 24±0.6 | 23±0.6 |

**^a^Used test strains:** *Escherichia coli* WKPM-M17 (EC), *Pseudomonas aeruginosa* GRP3 (VKPH B-82–5) (PA), *Bacillus subtilis* WT-A1 (BS)*, Salmonella typhimurium* WDCM 1754 (ST)*, Staphylococcus aureus* WDCM 5233 (SA), *Candida albicans* 174 (CA)*, Candida guilliermondii* HP-17 (CG).

**^b^PC** – positive control (gentamicin (10 μg ml^−1^) (for bacteria), nystatin 20 μg ml^−1^ (for yeasts)

All experiments were independently repeated three times. Average means with standard deviations are represented. Standard deviations were calculated with GraphPad Prism 5.03 (GraphPad Software, Inc.; USA) software.
